# Adolescent Self-Reported Recovery for Substance Use in Illinois: Statewide Representative Epidemiological Study

**DOI:** 10.2196/82792

**Published:** 2026-04-29

**Authors:** Douglas C Smith, Crystal A Reinhart, Shahana Begum, Tarjani Bhatt

**Affiliations:** 1 Center for Prevention Research and Development (CPRD), School of Social Work, University of Illinois Urbana-Champaign, 100 Trade Centre Drive, Suite 300, Champaign, IL, 61820, United States

**Keywords:** recovery, adolescents, substance use disorder, epidemiology, recovery capital

## Abstract

**Background:**

Although recovery is a central tenet of the US substance use disorder service delivery system, empirical research on youth recovery remains limited and underdeveloped. Notably, no population-based representative surveys, either in the United States or internationally, currently assess recovery status among secondary school–aged youth (aged 14-18 years). Consequently, little is known about how many youth identify as being in recovery or about their characteristics and needs.

**Objective:**

This study presents the first statewide representative estimate of adolescent self-reported recovery (ASR), derived from a large Midwestern state in the United States.

**Methods:**

We used data from the 2024 Illinois Youth Survey, a weighted, statewide representative survey of students from 8th, 10th, and 12th grades across Illinois. We examined the prevalence of ASR with a widely used single-item question, “Do you consider yourself to be in recovery?” The question was presented after an instruction directing students to consider only substance use when responding. We estimated the prevalence of ASR and conducted descriptive analyses to characterize this group.

**Results:**

Among the 6871 participating students from the 10th and 12th grades, the prevalence of ASR was 3.3% (95% CI 2.6%-4.1%). Among participants with ASR, 51.1% (118/231) were female, 39% (90/231) identified as Latino or Latina, 38.1% (88/231) identified as White, and 13% (30/231) identified as Black or African American. The average age of participants with ASR was 16.5 (SD 1.14) years. Participants with ASR were demographically diverse, and a little over half received free or reduced-price lunch.

**Conclusions:**

Findings suggest that financial recovery capital may be particularly important for participants with ASR. This study provides the first population-based estimate of the prevalence of ASR and underscores the importance of including recovery status in large-scale surveys to inform and strengthen recovery support systems.

## Introduction

Recovery is undoubtedly an organizing principle of the US substance use disorder (SUD) service delivery system. For instance, the US Surgeon General’s report has a dedicated chapter on recovery [1], and federal and state agencies have the word “recovery” appearing in their organization names (eg, the Substance Abuse and Mental Health Services Administration houses an Office of Recovery). Additionally, nationally representative epidemiological studies have measured substance use recovery among adults. On the basis of a study that used data from the National Survey of Drug Use and Health, about 8% of US adults consider themselves to be in substance use recovery [2].

Focusing on recovery as an organizing concept for care is part of a broader effort to frame SUD—a clinically diagnosable condition with substance use—as a treatable health condition. Additionally, the recovery movement seeks to destigmatize the millions of Americans who have recovered from SUD. Finally, there has been increasing attention on what supports are required to both initiate and maintain recovery [1].

Major advances have been made toward these goals. Historically, public stigma toward addiction and misperceptions about treatment effectiveness may have clouded health professionals’ willingness to treat patients with addiction. However, since the early 2000s, researchers have shown that treatment outcomes for people with SUD are similar to, and sometimes better than, those for other chronic health care conditions [3]. Major initiatives such as the implementation of the screening, brief intervention, and referral to treatment model in primary care have also moved SUD treatment into mainstream health care and improved SUD case identification [4]. However, the opioid crisis in the United States revealed a fissure in health care provision for SUD, with very low rates of efficacious medications being prescribed for persons with opioid use disorders [5].

Regarding stigma reduction, researchers have focused on identifying stigmatizing terms [6,7] that can be avoided by professionals. One clear term to avoid is “substance abuse,” which has been associated with professionals viewing persons with SUD as in need of punitive responses [7] and as dangerous [6]. Because of findings like these, some state agencies have removed “substance abuse” from their agency names. Another focus of destigmatizing recovery involves identifying potential workplace discrimination. For example, one national survey found that people remain leery of hiring individuals even when they are in recovery [8], signaling that additional work must be done to frame SUD as a treatable health condition.

Finally, there are many efforts to improve the infrastructure to maintain recovery once it is achieved. Two examples include the development of recovery community organizations and collegiate recovery programs, which both provide support for recovery maintenance [9,10]. While the former are community centers run by people in recovery through many programs (ie, antistigma events, support groups, and training of peer recovery specialists), the latter are specialized programs in universities designed to provide social support to college students (ie, support groups and sober tailgates). Additionally, many health care systems have integrated persons with lived experience into paraprofessional roles to help support those entering recovery [11]. All these efforts are part of an overall movement toward creating a recovery-oriented system of care that prizes lived experience, provides recovery support, and destigmatizes people with SUD.

However, with few exceptions, what is known about the pathways into and benefits of recovery is almost entirely based on adult research. Some progress has been made in adapting adult-focused models of recovery support for adolescents. For example, recovery high schools congregate adolescents who have entered recovery and could benefit from intense, structured social support [12,13]. Another model of adolescent recovery support is the alternative peer group model, which emphasizes sober recreation, peer support, and linkage to services [14]. While promising, both of these models are not widely available, which is likely driven by the fact that there are no nationally representative epidemiological data on the number of youth in recovery who may benefit from such supports [1].

The purpose of this study is to obtain a more accurate estimate of the prevalence of adolescent self-reported recovery (ASR) than that reported in prior studies. This could help policymakers better assess the need for structured recovery support for younger adolescents. This study used a weighted random sample of students from 10th and 12th grade in a Midwestern US state, which improves upon prior estimates. For example, to our knowledge, only 1 prior study estimated ASR, but it was not representative of the state from which the sample was drawn. It is estimated that 4.9% of 10th graders and 4.6% of 12th graders (roughly aged 16-18 years) may consider themselves to be in recovery [15]. In this study, we address a major weakness of the prior study by using 2024 data from the same statewide survey, which is representative. Thus, this study is the first statewide representative estimate of ASR to be conducted.

## Methods

### Ethical Considerations

Human subjects approval for the primary study was obtained from the authors’ institutional review board (University of Illinois; IRB24-0832), which included authorization to analyze and disseminate aggregated findings for secondary analyses such as this study. This study was approved for a waiver of parental consent, with parental notification and opt-out procedures in place. Schools were instructed not to survey students whose parents had opted them out of the survey and to provide such students with an alternative activity. However, the research team did not receive data on the number of students whose parents declined their participation in the survey.

### Study Description

The data used in this study come from the 2024 Illinois Youth Survey (IYS), a biennial survey administered to students in grades 8, 10, and 12. Participation was open to all schools in Illinois. Schools were recruited through a variety of mechanisms, including direct emails to principals and other school contacts from previous survey administrations, telephone calls, online newsletters and press releases, and conference exhibits (eg, the Illinois Principals’ Association conference). Those who opted to participate registered online and were sent instructions on distributing parent notifications, instructions on administering the survey across grades at the school, teacher or proctor instructions to be read aloud to students on survey day, and login information for the online survey. Schools administered the survey on a date of their choosing between January 2024 and June 2024, and students completed the survey on school-issued devices during scheduled classroom periods. Some schools, especially larger schools, are surveyed over a few days or a week.

### Data Validation

Multiple procedures were applied to ensure internal consistency and data quality [16]. Internal consistency was evaluated by cross-validating related items (eg, past 30-day vs past-year substance use). For instance, respondents who reported alcohol use in the past month but not in the past year were classified as inconsistent and the corresponding survey items were recoded as missing. Following these checks, 12.4% of surveys were invalidated. Surveys were removed if respondents (1) endorsed the use of a fictitious drug (1%), (2) completed fewer than 40% of survey items (9%), or (3) selected 2 of the lowest honesty options (2.8%). Some surveys met more than one exclusion criterion, resulting in overlap.

### Sampling Procedures and School Participation

While participation in the IYS was open to all schools, the data used for this study were gathered from schools randomly selected to comprise a statewide representative sample (refer to sampling procedures explained in subsequent sections). The sampling frame excluded very small schools (fewer than 16 students per grade) and all specialized institutions, such as alternative and charter schools, which may differ from traditional public schools in student composition.

During the 2024 administration cycle, a total of 10,490 surveys were collected from 154 sampled schools, including 74 middle schools and 80 high schools. Student participation rates varied across schools, with median participation rates of 81.5% (IQR 71.9%-86.5%) in middle schools and 55.3% (IQR 34.5%-69.2%) in high schools. This is consistent with participation rates in recent nationally representative surveys, which have dropped since the COVID-19 pandemic [17]. Participating schools included 60 from suburban Chicago, 29 from Chicago, 34 from other urban areas, and 31 from rural Illinois. To ensure adequate representation across grade-by-region strata, schools were oversampled at recruitment. Consequently, the target number of participating schools was achieved in most strata, and all participating schools were retained for analysis. Sampling weights were applied to account for differential probabilities of selection and participation.

The IYS uses a 2-stage, stratified cluster sampling design to generate a statewide representative sample of Illinois public schools serving students from 8th, 10th, and 12th grades. Schools were stratified by geographic region (Chicago, suburban Chicago, other urban or suburban counties, and rural counties) and grade level (8th, 10th, and 12th), yielding 12 strata to account for variation in student distribution across the state. In the first stage, schools were randomly selected within each stratum using a probability-proportional-to-size approach, accounting for differences in grade-level enrollment. In the second stage, all eligible students within selected schools were invited to participate, rather than sampling classrooms, to reduce logistical constraints and minimize within-class clustering. Within each participating school and grade, up to 50 completed surveys were randomly selected; schools with fewer than 50 respondents contributed all completed surveys [18-20]. This approach ensured equal selection probabilities across grades.

Survey weights were constructed to account for differential selection probabilities at the school and student levels. An additional absenteeism-adjustment factor was applied, giving greater weight to students with higher levels of school absence due to the known association between absenteeism and substance use [21]. Final weights were normalized to preserve the original sample size. When used with the specified survey design (including stratification), they yield estimates representative of the statewide public school population.

The 2024 IYS weighted dataset consisted of 10,490 students from 154 sampled schools. For this study, we focused on the subset of students (n=6986) from 10th and 12th grades who were administered the recovery status item. This study provides the first population-based estimate of the prevalence of ASR in Illinois. We hypothesized that the prevalence of ASR would be lower than that reported for adults in recovery.

### Measures

The IYS functions primarily as a prevention survey, with most items adapted from the Communities That Care Survey [22]. It includes a wide range of demographic measures, risk factors for initiating substance use, detailed substance use and consequence items, and a variety of other health and safety measures.

The primary variable of interest in this study was self-identified recovery status. Students were asked a single yes-or-no question, “Do you consider yourself to be in recovery?”, which has been used in prior adult research [2,19]. To avoid confusion with mental health recovery, this item appeared within a section clearly labeled: “The following questions are about recovery from substance use.” As done in other adult and adolescent research, immediately before the recovery item, students were asked, “Besides nicotine, did you used to have a problem with drugs or alcohol, but no longer do?” [2,15,19]. An affirmative response to this item is indicative of problem resolution but is not considered indicative of being in recovery.

### Analyses

All analyses accounted for the complex sampling design of the IYS and were conducted in both SPSS (version 31.0; IBM Corp) and Stata (version 19.1; StataCorp). Descriptive statistics, including prevalence estimates and 95% CIs, were calculated using the survey design and sampling weights to obtain design-adjusted SEs.

Missing data were minimal for demographic variables (<1.5%) and for the recovery item (3.5%). To evaluate the missing data mechanism, we conducted the Little Missing Completely at Random test on analytic variables, which did not indicate evidence against missing completely at random (*χ*²_50_=63.94; *P*=.09). However, given the strong conceptual and empirical links between recovery status and related substance use behaviors, multiple imputations were conducted under the more general missing-at-random assumption. The imputation model incorporated a broad set of alcohol-related and marijuana-related measures to improve the plausibility of missing at random and the quality of imputed recovery estimates.

Recovery prevalence estimates were calculated overall and by grade level. Descriptive characteristics of youth who identified as being in recovery are also presented.

### Analytic Sample

Among the 6986 students from 10th and 12th grades, we applied a consistency check to ensure the validity of self-reported recovery status. Students (115/6986, 1.6%) who indicated never having used substances on the age-of-first-use item but simultaneously endorsed being in recovery were classified as inconsistent and excluded from the analysis. This step was taken to improve the accuracy of the recovery prevalence estimates. After applying this exclusion, the final analytic, unweighted sample included 6871 students.

## Results

### Weighted Prevalence of Recovery by Grade

The weighted prevalence of youth who self-identified as being in recovery is presented in Table 1. Overall, 3.3% (231/6909; 95% CI 2.6%-4.1%) of students from 10th and 12th grades reported being in recovery, while 96.6% (6678/6909) reported not being in recovery. When examined by grade level, recovery prevalence was 3.3% (120/3592; 95% CI 2.4%-4.2%) among 10th graders and 3.3% (111/3317; 95% CI 2.4%-4.5%) among 12th graders. In both grades, more than 96% of students reported not being in recovery.

**Table 1.** Weighted prevalence of adolescent self-reported recovery (ASR) (N=6871).

Grade	Participants with ASR, n (%)	95% CI^a^	Adolescents not reporting recovery, n (%)
10th	120 (3.3)	2.4-4.2	3472 (96.6)
12th	111 (3.3)	2.4-4.5	3206 (96.6)

a 95% CIs correspond to the prevalence estimates of ASR and are based on pooled results from multiple imputed analyses.

### Weighted Prevalence of Recovery by Demographic Groups and Characteristics

Table 2 presents the weighted prevalence of ASR by demographic factors and characteristics of the participants with ASR. Among the 231 participants with ASR, the mean age was 16.5 (SD 1.14) years. Recovery prevalence was higher among female participants (n=118, 3.8%) than among male participants (n=110, 2.8%), with a small proportion preferring not to answer.

**Table 2.** Weighted prevalence and characteristics of adolescent self-reported recovery (ASR) (n=231)^a^*.*

Variables	Participants with ASR, n (%)	Prevalence of ASR, % (95% CI)
Sex		
Female	118 (51.1)	3.8 (2.8-4.8)
Male	110 (47.6)	2.8 (2.0-3.5)
Prefer not to answer	3 (1.3)	—^b^
Race		
White	88 (38.1)	2.2 (1.5-3.0)
Black or African American	30 (13.0)	4.2 (1.8-6.5)
Latino or Latina	90 (39.0)	5.3 (3.5-7.2)
Asian American	10 (4.3)	—
Other	13 (5.6)	—
Grade		
10th	120 (52.1)	3.3 (2.4-4.2)
12th	111 (47.9)	3.3 (2.4-4.5)
Region		
Suburban Chicago	123 (53.2)	3.2 (2.5-4.0)
Chicago	40 (17.3)	4.3 (1.7-6.8)
Other urban or suburban areas	33 (14.3)	2.3 (1.2-3.5)
Rural areas	35 (15.2)	4.9 (2.9-6.9)
Free or reduced-price lunch		
Free lunch	116 (50.2)	4.3 (3.2-5.5)
Reduced-price lunch	13 (5.6)	3.4 (1.3-5.4)
Neither	102 (44.2)	2.4 (1.5-3.3)

a Mean age for participants with ASR was 16.5 (SD 1.14) years.

b Prevalence and CIs are not shown for cells with small sample sizes due to limited precision.

By race, Latino or Latina youth had the highest prevalence of ASR (5.3%), followed by Black or African American youth (4.2%) and White youth (2.2%). Sample sizes for Asian American and other racial groups were too small to support reliable estimates. Although White youth had a lower prevalence of ASR, they accounted for a substantial share of the total ASR population (88/231, 38.1%) due to their larger representation in the overall student population. In contrast, Latino or Latina youth, who had the highest prevalence of ASR (5.3%), comprised a disproportionately large share of the ASR population (90/231, 39%) relative to their representation in the student population.

The prevalence of ASR was comparable across grades, with estimates of 3.3% among 10th graders and 3.3% among 12th graders, and the proportion of participants with ASR was approximately evenly split between the two grades (120/231, 52.1% and 111/231, 47.9%, respectively).

Regional differences in prevalence of ASR were observed, with the highest prevalence in rural areas (4.9%), followed by Chicago (4.3%), suburban Chicago (3.2%), and other urban or suburban areas (2.3%). Among all participants with ASR, most were from suburban Chicago (123/231, 53.2%).

Regarding socioeconomic status, as indicated by free or reduced-price lunch status, the highest prevalence of ASR was among those receiving free lunch (4.3%), followed by those receiving reduced-price lunch (3.4%). The prevalence of ASR was lowest among youth not receiving any lunch assistance (2.4%). Approximately half (116/231, 50.2%) of all participants with ASR received free lunch.

## Discussion

### Principal Findings

In this Midwestern US state, 3.3% of adolescents self-reported being in recovery. Based on the total number of students in the 10th and 12th grades in Illinois, that equates to about 4375 tenth graders and 4455 twelfth graders. Comparably, about 4% to 8% of adults in the United States indicate that they are in recovery [2,23]. This is consistent with our hypothesis that adolescent self-reported recovery would be lower than estimates for adults, owing to fewer years of disordered use and fewer opportunities to make lifestyle changes associated with being in recovery.

Furthermore, we note that the overall prevalence rate of ASR here (3.3%) is lower than that previously reported for adolescents (4.3%) in the same state [15]. This could be because this study used a statewide representative sample, whereas the prior sample did not. Additionally, this study used more stringent data screening, excluding students who reported “never using” substances on items about age of onset but also reported being in recovery. In this study, this exclusion criterion lowered the overall prevalence estimate from 5.1% to 3.3%, a considerable difference. This finding highlights the importance for researchers measuring prevalence of ASR to incorporate validity checks.

On the basis of this and other work, we urge national epidemiological studies to start measuring youth recovery. First, these estimates will help improve the planning and development of recovery support services for adolescents. For example, recovery high schools, specially designed to support youth in recovery, are only available in some areas. These findings may support the plausibility of establishing a recovery high school in Chicago or its suburbs. Second, compared to small clinic-based studies of adolescent recovery, studies based on large epidemiological designs can capture a wide variety of recovery pathways, including nonabstinent recovery. There are many definitions of recovery [1]. Regrettably, we know little about nonabstinent youth in recovery [24], although small studies suggest that many youth are interested in this pathway [25].

Third, asking about youth recovery on epidemiological surveys may lead to new insights through the identification of recovery hot spots; that is, it would enable researchers to evaluate whether systems-level initiatives in recovery promotion (ie, recovery community organizations) are working. Emerging research has shown that county-level recovery ecosystems, measured by the presence of treatment, support groups, and recovery-friendly policies, predict ASR indirectly through parent and peer disapproval [26]. Given the need for community recovery capital [27] to support youth recovery, these findings support the validity of the item we used here and in prior research to measure recovery [15].

Finally, youth recovery may be a critical prevention indicator in the future. Although recovery is generally thought of as a treatment outcome in existing patient placement criteria for SUDs, youth recovery could also be conceptualized as a prevention indicator in the broader health context; that is, participants with ASR could reasonably be expected to experience better health outcomes in adulthood due to lifestyle choices associated with earlier recovery.

### Limitations

The following limitations apply to this study. First, the data were self-reported by adolescents, and it is unclear whether all students fully grasped the concept of recovery, despite the introductory heading clarifying that questions referred to substance use. While the survey contractor did not inquire whether schools used monitoring of school devices (ie, GoGuardian), schools were instructed to provide a private space for students to participate and were informed that the survey was anonymous. Research shows that group-administered computerized surveys can reduce reporting of socially undesirable topics due to concerns about confidentiality, even with anonymity assurances [28]. Nevertheless, it is possible that surveillance software used by schools resulted in underreporting. Future studies should track and transparently report whether schools use monitoring software. This would enable better sensitivity testing of its impact on reporting sensitive information.

Second, estimates for 12th graders in the Chicago strata may be less reliable due to small sample sizes at randomized schools and lower student participation. However, as the majority of students in Illinois (223,547/406,719, 55%) live in suburban school districts, the slightly higher recovery estimates in Chicago may only result in a slight upward bias in the statewide prevalence rate. Third, as this survey only included students who were present in school on the days of survey administration, it is a limitation that it does not include students who were absent or had left school. We do not know how this would affect ASR estimates. While absenteeism would affect substance use estimates [21], participants with ASR may experience improvements in school attendance. Future research should clarify this phenomenon. Fourth, these findings are specific to Illinois public school 10th-grade and 12th-grade students and should not be extrapolated to other states or countries. Future research is needed to assess whether youth recovery prevalence differs nationally or internationally.

Fifth, because the data source is a prevention survey, some variables of interest are unavailable (eg, recovery supports and SUD diagnoses). However, we note that SUD screening scores were elevated among participants with ASR in our prior research [15], possibly supporting the validity of our single-item measure of recovery for adolescents. Finally, the statewide representative sample excluded small schools or specialized institutions, including alternative and charter schools. In sensitivity analyses, we found that alternative schools (in our nonweighted sample) had a higher prevalence of ASR. The exclusions of both absent students and students from alternative schools necessitate additional research on marginalized students. Such students are often less likely to attend school and are also overrepresented in alternative schools [29,30]. However, the overall impact on the state prevalence of the ASR estimate would be low because there are so few alternative schools. Nevertheless, although the data are representative of mainstream public schools, the prevalence estimates reported here may not fully generalize to all youth.

### Conclusions

Recovery is an organizing concept of the US SUD service delivery system, which is why it is peculiar that there is no national estimate of youth recovery. This statewide representative survey of a large Midwestern US state found that 3% of youth in 10th and 12th grades considered themselves to be in recovery. We strongly encourage the adoption of a recovery status item on national and international epidemiological studies involving adolescents under the age of majority. Doing so may lead to better recovery support planning, a better understanding of nonabstinent recovery pathways, more research on recovery hot spots, and improved understanding of whether youth recovery functions as both a treatment outcome and a protective factor for future health status.

## Abbreviations

ASR: adolescent self-reported recovery
IYS: Illinois Youth Survey
SUD: substance use disorder
